# Aficamten in symptomatic obstructive hypertrophic cardiomyopathy: the FOREST-HCM long-term study

**DOI:** 10.1093/eurheartj/ehaf1085

**Published:** 2026-03-05

**Authors:** Albree Tower-Rader, Ahmad Masri, Michael E Nassif, Theodore P Abraham, Roberto Barriales-Villa, Lubna Choudhury, Robert M Cooper, Perry M Elliott, Martin S Maron, Iacopo Olivotto, Artur Oreziak, Anjali Tiku Owens, Scott D Solomon, Stephen B Heitner, Daniel L Jacoby, Stuart Kupfer, Xueli Liu, Fady I Malik, Chiara Melloni, Tyrell J Simkins, Jenny Wei, Sara Saberi

**Affiliations:** Cardiology Division, Department of Medicine, 55 Fruit St, Yawkey 5B, Massachusetts General Hospital, Boston, MA 02114, USA; Division of Cardiovascular Medicine, Oregon Health and Science University, Portland, OR, USA; Division of Cardiology, University of Missouri Kansas City Healthcare Institute for Innovations in Quality and Saint Luke’s Mid America Heart Institute, Kansas City, MO, USA; Divison of Cardiology, University of California San Francisco, San Francisco, CA, USA; Complexo Hospitalario Universitario A Coruña, INIBIC, CIBERCV-ISCIII, A Coruña, Spain; Divison of Cardiology, Northwestern University Feinberg School of Medicine, Chicago, IL, USA; Department of Cardiology, Liverpool John Moores University, Liverpool, UK; Barts Heart Centre, University College London, London, UK; Cardiovascular Division, Lahey Hospital and Medical Center, Burlington, MA, USA; Meyer Children’s Hospital, Istituto di Ricovero e Cura a Carattere Scientifico (IRCCS), Florence, Italy; 1st Department of Arrhythmia, National Institute of Cardiology, Warsaw, Poland; Division of Cardiovascular Medicine, University of Pennsylvania Perelman School of Medicine, Philadelphia, PA, USA; Cardiovascular Division, Brigham and Women’s Hospital, Harvard Medical School, Boston, MA, USA; Cytokinetics, Incorporated, South San Francisco, CA, USA; Cytokinetics, Incorporated, South San Francisco, CA, USA; Cytokinetics, Incorporated, South San Francisco, CA, USA; Cytokinetics, Incorporated, South San Francisco, CA, USA; Cytokinetics, Incorporated, South San Francisco, CA, USA; Cytokinetics, Incorporated, South San Francisco, CA, USA; Cytokinetics, Incorporated, South San Francisco, CA, USA; Cytokinetics, Incorporated, South San Francisco, CA, USA; Division of Cardiovascular Medicine, University of Michigan, Ann Arbor, MI, USA

**Keywords:** Aficamten, Efficacy, Hypertrophic cardiomyopathy, Safety, Open-label extension

## Abstract

**Background and Aims:**

Aficamten is a next-in-class, oral selective cardiac myosin inhibitor that ameliorates hypercontractility in hypertrophic cardiomyopathy (HCM). This study assessed the safety and efficacy of extended aficamten treatment in symptomatic obstructive HCM (oHCM).

**Methods:**

Patients completing a parent aficamten study were eligible to enrol in FOREST-HCM (NCT04848506), an open-label study evaluating long-term aficamten treatment.

**Results:**

Patients with oHCM (*N* = 296; mean age ±SD 61 ± 12.3 years, 44.3% female) enrolled between May 2021 and August 2024. Cumulative exposure was 352 patient-years; median follow-up 51.6 (IQR 41.5, 70.8) weeks. At Weeks 12 and 96, aficamten reduced Valsalva left ventricular outflow tract gradient by 56 ± 43 and 62 ± 33 mmHg from baseline (both *P* < 0.0001), with minimal reduction in left ventricular ejection fraction (LVEF) (−3% ± 6% and −5% ± 5%); 69% and 93% of participants had at least one NYHA class improvement; Kansas City Cardiomyopathy Questionnaire–Clinical Summary Score improved by 15 ± 16 and 16 ± 16 points. Treatment-emergent serious adverse events (TESAEs) occurred in 36 (12.2%) patients; no deaths, heart failure, or events considered related to aficamten were reported. One (0.3%) patient terminated therapy due to a TESAE (ischemic colitis). LVEF<50% occurred in 10 (3.4%) patients [exposure-adjusted incidence rate (EAIR): 2.9 per 100 patient-years] with 2 having non-serious mild/moderate dyspnoea. No treatment interruptions for LVEF<50%, and no events of LVEF<40% occurred. New-onset atrial fibrillation occurred in seven (2.4%) patients (EAIR 2.0 per 100 patient-years).

**Conclusions:**

Extended aficamten treatment in patients with symptomatic oHCM yielded early and sustained hemodynamic and clinical responses with low incidences of new-onset atrial fibrillation and LVEF<50%.

## Structured Graphical Abstract

**Structured Graphical Abstract:**
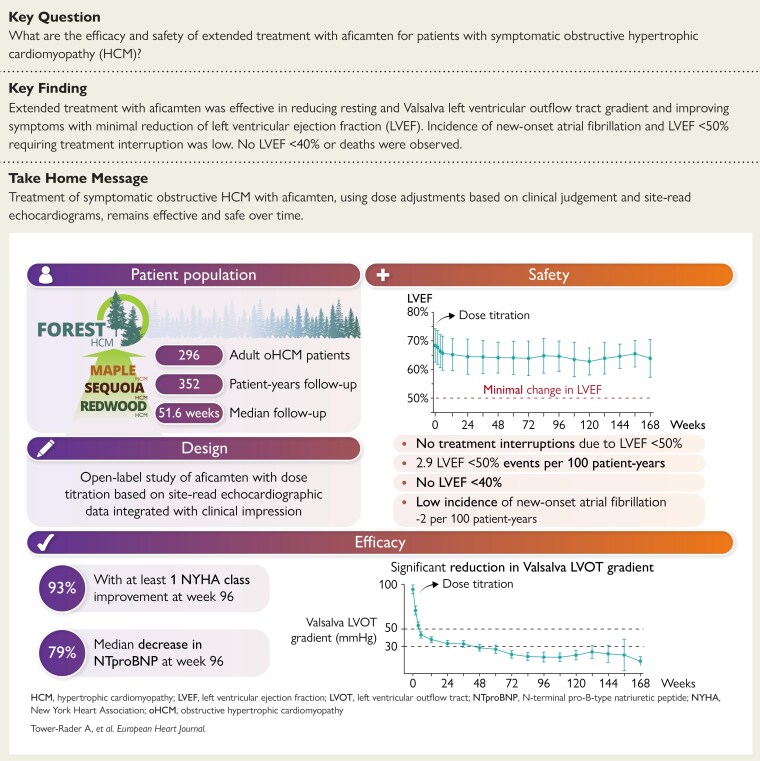

**See the editorial comment for this article ‘Long-term safety and efficacy of aficamten in the evolving era of myosin inhibition for obstructive hypertrophic cardiomyopathy’, by L.R. Lopes, https://doi.org/10.1093/eurheartj/ehag059.**

## Introduction

Hypertrophic cardiomyopathy (HCM) is characterized by increased left ventricular (LV) wall thickness, hypercontractility, and diastolic dysfunction with resting or provocable LV outflow tract (LVOT) obstruction in up to two-thirds of patients, leading to symptoms of exercise intolerance, dyspnoea, angina, and presyncope or syncope.^1^ Historically, treatment options have been limited to non-vasodilating beta-blockers, non-dihydropyridine calcium channel blockers, and septal reduction therapy (SRT).^1,2^ Cardiac myosin inhibitors represent the first new therapeutic and potentially disease-modifying intervention for patients with symptomatic obstructive HCM (oHCM) since the development of alcohol septal ablation in the 1990s.^3^ Following US Food and Drug Administration approval of mavacamten in 2022, cardiac myosin inhibitors were recently added to clinical practice guidelines as second-line therapy for patients with oHCM who remain symptomatic despite treatment with either a non-vasodilating beta-blocker or non-dihydropyridine calcium channel blocker.^1,2^

Aficamten is a next-in-class, oral, selective cardiac myosin inhibitor with a half-life of 3.4 days (reaching steady-state within 2 weeks), a shallow pharmacokinetic–pharmacodynamic relationship, and a lack of common clinically relevant cytochrome P450 drug–drug interactions.^4,5^ Prior phase 2 (REDWOOD-HCM [Randomized Evaluation of Dosing With CK-3773274 in Obstructive Outflow Disease in HCM], NCT04219826) and phase 3 (SEQUOIA-HCM [Safety, Efficacy, and Quantitative Understanding of Obstruction Impact of Aficamten in HCM], NCT05186818) clinical trials demonstrated robust efficacy and safety features of aficamten with improvements seen in exercise capacity, health status, symptoms, haemodynamics, and reduced indication for SRT with 10 and 24 weeks of aficamten treatment.^6,7^ FOREST-HCM (Follow-up, Open-Label, Research Evaluation of Sustained Treatment with Aficamten in HCM; NCT04848506) was designed to study the safety and efficacy of long-term aficamten treatment, with a study protocol replicating the real-world experience by implementing dose adjustments based on site-read echocardiograms and allowing for clinical judgement to be integrated into decision-making. Here, we report comprehensive data regarding efficacy and safety over extended follow-up.

## Methods

### Study design

FOREST-HCM is an ongoing multicentre, open-label study of aficamten for adult patients (aged ≥18 years) with HCM and LVEF ≥55% who completed an aficamten parent study. Patients who completed REDWOOD-HCM, SEQUOIA-HCM, or MAPLE-HCM (Phase 3 Trial to Evaluate the Efficacy and Safety of Aficamten Compared to Metoprolol Succinate in Adults With Symptomatic oHCM; NCT05767346) were eligible for enrolment during the current analysis period. The inclusion/exclusion criteria and study design for the parent studies have been published.^6–8^ Of note, MAPLE-HCM was ongoing at the time of data cutoff; thus, not all patients enrolled in MAPLE-HCM were yet eligible for FOREST-HCM. For the purposes of this analysis, only patients with oHCM were included.

Per parent study design, patients completed a 4-week wash-out from their study drug (aficamten, metoprolol, or placebo, dependent on investigational drug assignment) prior to end of the parent study. During FOREST-HCM, patients attended regularly scheduled visits that included clinical assessments, patient-reported outcome questionnaires, laboratory assessments, and echocardiograms (see Supplementary data online, *Figure S1*). Genetic testing was not required per protocol; thus data regarding sarcomere gene mutation status was reported if available (see Supplementary data online, *Table S1*). Patients were defined as complete responders if they were New York Heart Association (NYHA) functional class I and had Valsalva LVOT gradient <30 mmHg. Patients were eligible for SRT if they had NYHA functional class ≥ III and resting or Valsalva LVOT gradient ≥50 mmHg at baseline. Enrolment started on 28 May 2021 and is ongoing. This interim analysis used the most recent data cutoff of 31 August 2024. The protocol for FOREST-HCM was approved by local or central Institutional Review Boards for each site, and patients provided written informed consent. The trial was conducted following guidance from the Declaration of Helsinki and International Council for Harmonisation Good Clinical Practice Guidelines.

### Aficamten dose titration

In brief, aficamten was started at 5 mg daily, and patients were eligible for a 5 mg dose increase if site-read post-Valsalva LVOT gradient was ≥30 mmHg and LVEF ≥55%. The titration period consisted of visits at approximately weeks 2, 4, and 6. At any visit after the titration period, the dose could be increased if the patient met the echocardiographic eligibility criteria. Up-titration of aficamten was not mandated for eligible patients. Investigators interpreted all echocardiographic data used for titration purposes and integrated their clinical impression into dose-adjustment decisions (e.g. an investigator might choose not to increase the aficamten dose if the LVOT gradient was marginally above threshold but the patient was otherwise asymptomatic). The maximal dose in the original protocol was 15 mg, which was subsequently increased to 20 mg daily with a protocol amendment in December 2021. The dose was maintained for patients with an LVEF ≥50% who were not eligible for an increase or who had reached the maximum dose of 20 mg. The dose was decreased to the prior lower dose if LVEF was 40%–49%. If LVEF was <40% at any time during the study, or <50% before or at visit 2 (week 2), then aficamten was interrupted and could potentially be resumed after LVEF was ≥55% following discussion with the medical monitor. A subsequent protocol amendment enacted after this analysis allows for greater flexibility in titration and less frequent monitoring. Echocardiograms were subsequently reviewed by the Cardiovascular Imaging Core Laboratory (Brigham and Women’s Hospital, Boston, MA, USA) for assessment of additional echocardiogram variables, but the core laboratory data were not used to inform dose titration.

### Outcomes

The primary objective of FOREST-HCM is to assess the safety and tolerability of aficamten on the basis of incidences of treatment-emergent adverse events (TEAEs), treatment-emergent serious adverse events (TESAEs), and dose reductions due to LVEF <50%. A TEAE was defined as any unfavourable medical occurrence in a patient administered aficamten. A TESAE was defined as any unfavourable medical occurrence which resulted in significant disability or incapacity, required hospitalization or prolongation of a hospitalization, was life-threatening, or resulted in death. Secondary objectives for FOREST-HCM were change from baseline in LVOT gradient in patients with oHCM and time to prespecified reductions in LVOT gradient (i.e. resting LVOT <30 mmHg, Valsalva LVOT <50 mmHg). Exploratory objectives include assessing the long-term effects of aficamten on cardiac structure and function, eligibility for SRT, cardiac biomarkers (N-terminal pro-B-type natriuretic peptide [NT-proBNP] and high-sensitivity troponin I [hsTnI]), physician-assessed NYHA functional class, and patient-reported health status using the Kansas City Cardiomyopathy Questionnaire (KCCQ).^9^ As the site-determined LVEF and peak LVOT gradients were used for dose selection, they were also used in these analyses; however, other echocardiographic variables are as reported by the core lab. The Kansas City Cardiomyopathy Questionnaire (KCCQ) assesses a patient’s symptom frequency, burden, and impact on their daily life, with the KCCQ clinical summary score (KCCQ-CSS) encompassing symptom, physical, and social function scales.^10^ The KCCQ is scored from 0 to 100, with a higher score corresponding with better health status. A change in KCCQ score of 5 points is a clinically meaningful small change, whereas a change ≥20 points is a large change in patients with HCM.^10,11^ These metrics were assessed at baseline and each study visit. Cardiopulmonary exercise testing was not included in the study protocol.

### Statistical analysis

The safety population consisted of all patients who received at least one dose of aficamten. The efficacy population consisted of all enrolled patients who received aficamten at baseline and at least one post-baseline assessment. The last follow-up for patients was defined as the last study visit prior to data cutoff. Four patients were included in the safety population but not the efficacy population: two for whom only baseline information had been collected at the time of data cutoff and two from a site closed for violations of good clinical practice. Due to the exploratory nature and open-label study design, no formal hypothesis testing, formal sample size calculations or multiplicity adjustments were performed. Categorical variables are reported by number and percentages, whereas continuous variables are reported as mean with standard deviation (SD) or median with interquartile range (IQR; defined as Q1 = 25th percentile and Q3 = 75th percentile) when not normally distributed. For TEAEs, we report both the number of patients with TEAEs and persons with event per patient-years. Exploratory efficacy analyses were conducted using mixed model for repeated measures at a two-sided nominal *α* = 0.05. *P*-values are unadjusted for multiplicity and interpreted descriptively, and the corresponding 95% confidence intervals (CIs) are likewise nominal. SAS Enterprise Guide Version 8.3 was used for statistical data analysis.

## Results

### Baseline patient characteristics

At the time of interim data cutoff (31 August 2024), 296 patients with oHCM were enrolled in FOREST-HCM and had received at least one dose of aficamten. The first patient was enrolled on 28 May 2021. The majority of the 366 (81%) patients who completed a parent study and were eligible for FOREST-HCM were enrolled. The overall population included 45 of 54 (83.3%) patients from REDWOOD-HCM (Cohorts 1–3), 222 of 282 (78.7%) patients from SEQUOIA-HCM (excluding China), and 29 of 30 (96.7%) patients who completed MAPLE-HCM at the time of data cutoff. Patients enrolled in SEQUOIA-HCM in China participated in a separate long-term extension study instead of FOREST-HCM. The mean duration from the end of the parent study to the first dose in FOREST-HCM was 34.7 ± 17.6 weeks for patients who completed REDWOOD-HCM, 10.1 ± 5.3 weeks for patients who completed SEQUOIA-HCM, and 7.1 ± 1.6 weeks for patients who completed MAPLE-HCM.

Baseline patient demographics and clinical characteristics are summarized in *Table 1*. The mean ± SD age at enrolment was 61.0 ± 12.3 years, and 131 (44.3%) were female. A documented family history of HCM or the presence of a pathogenic, or likely pathogenic variant in an HCM-associated gene was reported in 101 (34.1%) patients. Frequency of reported sarcomeric gene mutations may be found in Supplementary data online, *Table S1*. At the time of enrolment, 248 (83.8%) patients were receiving background pharmacologic therapy. The most frequently co-administered drug regimen was beta-blocker monotherapy (*n* = 147, 49.7%), followed by non-dihydropyridine calcium channel blocker monotherapy (*n* = 51, 17.2%) and combination therapy involving two or more agents (*n* = 49, 16.6%). Disopyramide was included in the background therapy of 44 (14.9%) patients either as monotherapy or in combination with a beta-blocker or non-dihydropyridine calcium channel blocker. A total of 48 (16.2%) patients were not on background therapy. Of these, 19 (40%) patients transitioned from the MAPLE-HCM study in which patients were randomized to either metoprolol or aficamten as monotherapy and underwent post-treatment washout. The other 29 patients were not on background therapy either because of intolerance or contraindications.

**Table 1:** Baseline characteristics

Characteristic	
Patients, *n*	296
Age, years, mean ± SD	61.0 ± 12.3
Female sex, *n* (%)	131 (44.3)
Race, *n* (%)	
White	278 (93.9)
African American or Black	5 (1.7)
Asian	9 (3.0)
Other	4 (1.3)
BMI, kg/m^2^, mean ± SD	28.9 ± 4.2
Medical history, *n* (%)	
Atrial fibrillation	51 (17.2)
Paroxysmal/persistent	46 (15.5)
Permanent	5 (1.7)
Implantable cardioverter-defibrillator	46 (15.5)
Hypertension	143 (48.3)
Known HCM-causing gene mutation or positive family history, *n* (%)	101 (34.1)
Heart rate, b.p.m., mean ± SD	65.0 ± 10.8
Systolic blood pressure, mmHg, mean ± SD	124.2 ± 15.95
Diastolic blood pressure, mmHg, mean ± SD	73.0 ± 10.66
NYHA class, *n* (%)	
I	10 (3.4)
II	168 (56.8)
III	118 (39.9)
KCCQ-CSS, mean ± SD	70.5 ± 19.4
Background HCM therapy, *n* (%)	
β-Blocker monotherapy	147 (49.7)
Non-dihydropyridine calcium channel blocker monotherapy	51 (17.2)
Disopyramide monotherapy	1 (0.3)
≥2 medications	49 (16.6)
β-Blocker and CCB	6 (2.0)
β-Blocker and disopyramide	37 (12.5)
CCB and disopyramide	4 (1.4)
β-Blocker, CCB, and disopyramide	2 (0.7)
None	48 (16.2)
NT-proBNP, pg/mL, median [Q1, Q3]	776.5 [348.5, 1531.5]
hsTnI, ng/L, median [Q1, Q3]	11.2 [5.8, 19.8]
LVEF^a^, %, mean ± SD	68.3 ± 5.8
Resting LVOT-G^a^, mmHg, mean ± SD	56.4 ± 37.3
Valsalva LVOT-G^a^, mmHg, mean ± SD	94.1 ± 42.5

^a^Site-read baseline echocardiogram.

β-Blocker, beta-blocker; BMI, body mass index; CCB, calcium channel blocker; HCM, hypertrophic cardiomyopathy; hsTnI, high-sensitivity troponin I; KCCQ-CSS, Kansas City Cardiomyopathy Questionnaire clinical summary score; LVEF, left ventricular ejection fraction; LVOT-G, left ventricular outflow tract gradient; NYHA, New York Heart Association; NT-proBNP, N-terminal pro-B-type natriuretic peptide; Q, quartile; SD, standard deviation.

At baseline, mean LVEF was 68 ± 6%, mean resting LVOT gradient was 56 ± 37 mmHg, and post-Valsalva LVOT gradient was 94 ± 43 mmHg. Patients were symptomatic at baseline, with mean KCCQ-CSS of 70.5 ± 19.4 points, and 40% were NYHA class III. Median NT-proBNP was 776.5 pg/mL (IQR 348.5, 1531.5 pg/mL), and median hsTnI was 11.2 ng/L (IQR 5.8, 19.8 ng/L).

### Treatment duration

Total cumulative exposure for the 296 patients within FOREST-HCM consisted of 352 patient-years over a median follow-up of 51.6 (IQR 41.5, 70.8) weeks. A total of 173 patients completed week 48 follow-up, 44 patients completed week 96 follow-up, and 3 patients reached week 168.

### Dosage

A total of 292 patients had at least one post-baseline assessment and opportunity for dose titration. At the end of the titration period (week 12), aficamten dose distribution was as follows: 18 (6.4%) patients on 5 mg, 54 (19.3%) patients on 10 mg, 95 (33.9%) patients on 15 mg, and 113 (40.4%) patients on 20 mg. After the titration period, doses were overall stable for individual patients with extended follow-up. Patients who underwent down-titration are discussed in the section on Safety.

### Efficacy

#### Haemodynamic response

**Figure 1:**
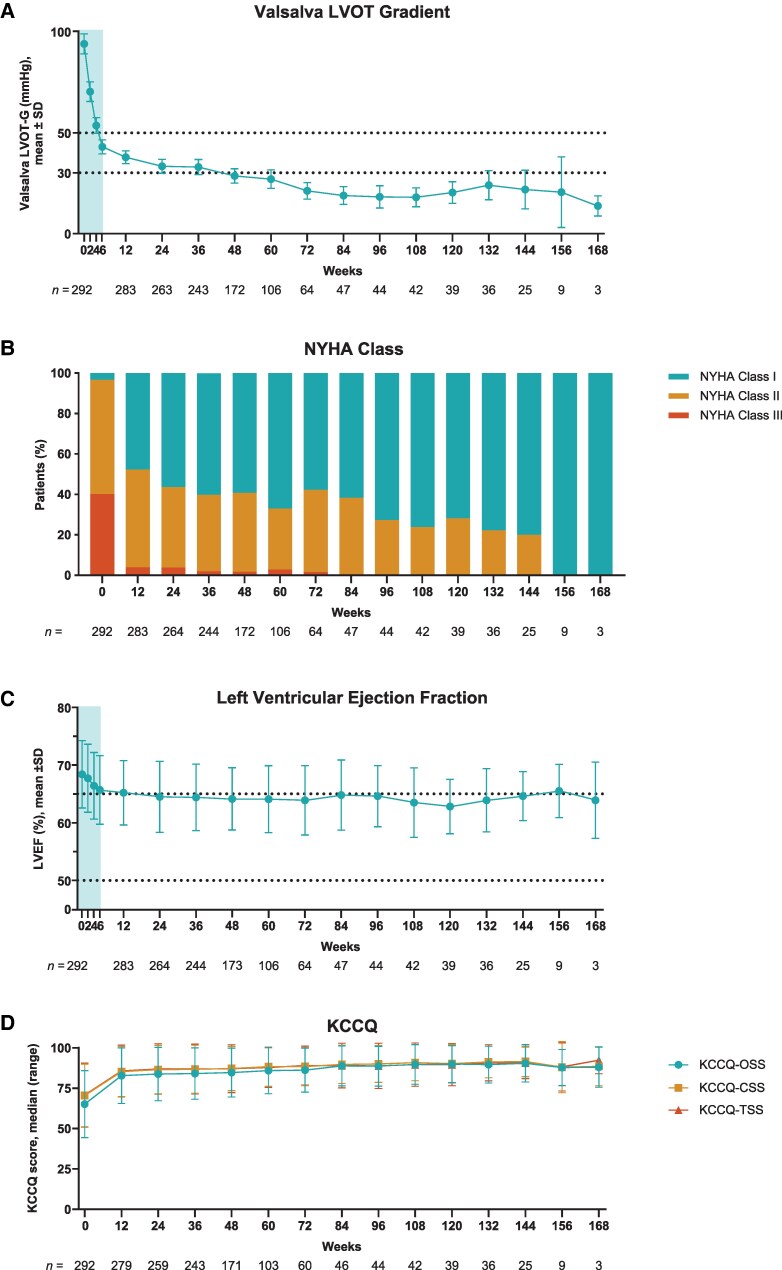
(*A*) Valsalva LVOT gradient, (*B*) NYHA class, (*C*) LVEF, and (*D*) KCCQ scores with treatment with aficamten up to week 168 follow-up. Grey shaded area in panels *A* and *C* represents the titration phase. KCCQ-CSS, Kansas City Cardiomyopathy Questionnaire clinical summary score; KCCQ-OSS, Kansas City Cardiomyopathy Questionnaire overall summary score; KCCQ-TSS, Kansas City Cardiomyopathy Questionnaire total symptom score; LVOT-G, left ventricular outflow tract gradient

Treatment with aficamten resulted in rapid and substantial reductions in mean resting and Valsalva LVOT gradients by week 12 (−40 ± 35 mmHg and −56 ± 43 mmHg, respectively, *n* = 283). This change was sustained throughout treatment, with reductions of 44 ± 37 mmHg and 64 ± 42 mmHg (*n* = 172) at week 48 and 42 ± 32 mmHg and 62 ± 33 mmHg (*n* = 44) at week 96 for resting and Valsalva LVOT, respectively (all *P* < .0001) compared with baseline. At week 12, 49.1% of patients had resting and Valsalva LVOT gradients <30 mmHg, with a greater proportion of patients achieving resting and Valsalva LVOT gradients <30 mmHg at week 48 and week 96 (61% and 84.1%, respectively). The haemodynamic improvement was accompanied by a small reduction in mean LVEF relative to baseline at week 12 (−3 ± 6%, *n* = 283), which was stable throughout treatment (−4 ± 6% at week 48, *n* = 173; −5 ± 5% at week 96, *n* = 44) (*P* < .0001 for all) (*Figure 1*).

#### Symptomatic response

By week 12, 195 (68.9%, *n* = 283) patients reported at least one NYHA class improvement from baseline that persisted at each visit after week 12 [139 (80.8%, *n* = 172) patients at week 48, 41 (93.2%, *n* = 44) patients at week 96]. At week 12, 135 (47.7%, *n* = 283) patients were asymptomatic (NYHA class I), with only 11 (3.9%, *n* = 283) patients reporting more than mild symptoms (NYHA class III–IV), representing an approximate 10-fold reduction relative to the baseline of 117 (39.9%, *n* = 292) (*Figure 1*). By week 48, 102 (59.3%, *n* = 172) patients had converted to NYHA class I, and, by week 96, 32 (72.7%, *n* = 44) had become asymptomatic. Patients reported a mean KCCQ-CSS improvement of 15.0 ± 16.4 points by week 12 (70.5 ± 19.4 points to 85.3 ± 15.5 points, *P* < .0001; *Figure 1*), and 31.9% experienced a large improvement (≥20 points) at each visit after week 12. These improvements were sustained with extended treatment, with mean improvement in KCCQ-CSS of 15.7 ± 16.3 points at week 48 and 16.4 ± 16 points at week 96 compared with baseline (both *P* < .0001).

### Clinical response

By week 12, 23.7% (67/283) of patients were complete responders and that proportion increased at subsequent visits with 40.5% (70/172) of patients at week 48 and 62% (28/44) of patients at week 96 experiencing a complete response. There were three (1%) patients who did not have improvement in Valsalva LVOT gradients or NYHA class at time of the last assessment before the data cutoff. All three of these patients had transient reductions in Valsalva LVOT gradients and NT-proBNP and achieved 20 mg dose without change in NYHA class. All three remained in the study (see Supplementary data online, *Table S2*).

### SRT eligibility

**Figure 2:**
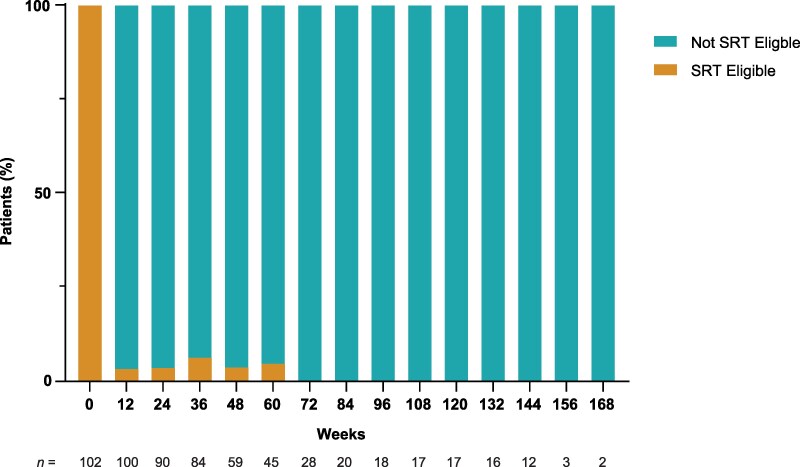
Eligibility for septal reduction therapy with treatment with aficamten up to week 168 follow-up

Of the 101 patients (34.1% of the overall cohort) who were SRT-eligible at baseline, only three (3%, *n* = 99) patients continued to meet the definition for SRT eligibility by week 12; two (3.4%, *n* = 58) patients were eligible at week 48, and no one was eligible by the week 96 visit (*Figure 2*). As noted in the Safety section, there was one patient who terminated the study and underwent septal myectomy at week 52 and one who terminated the study due to persistent elevated LVOT gradients at week 36 despite treatment with 20 mg aficamten.

### Biomarkers

**Figure 3:**
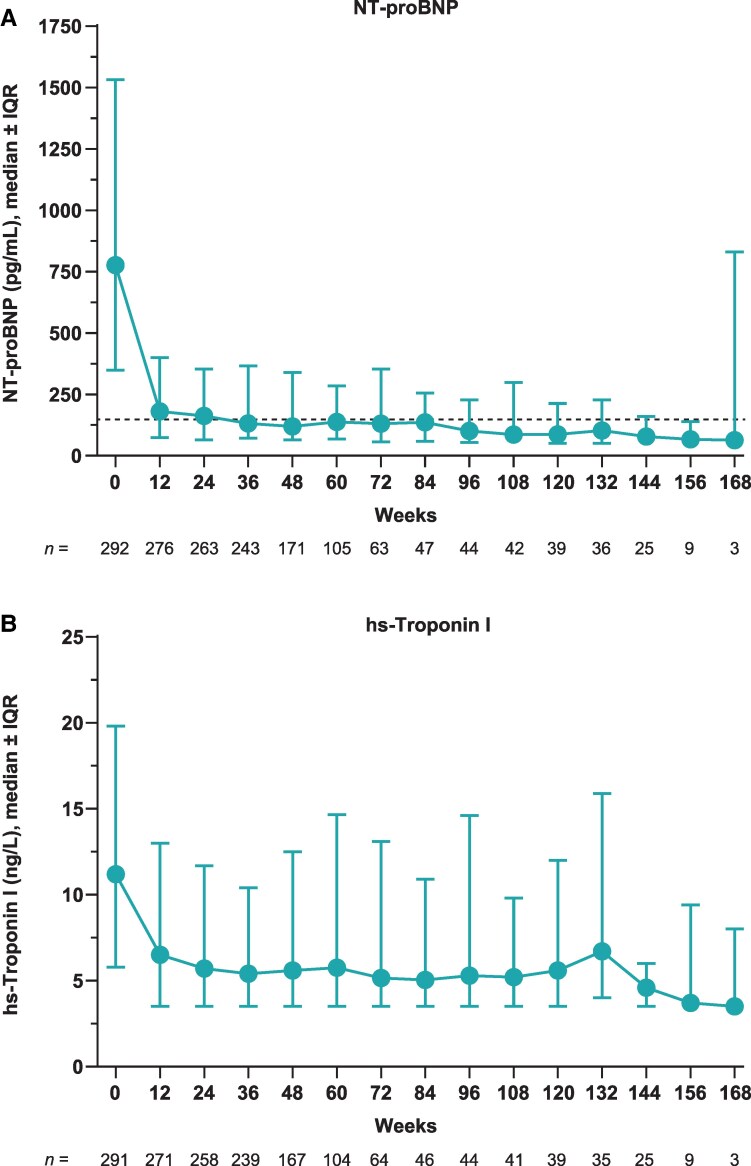
(*A*) NT-proBNP and (*B*) hs-troponin I with treatment with aficamten up to week 168 follow-up. NT-proBNP, N-terminal pro-B-type natriuretic peptide; hs, high-sensitivity

Substantial early reductions in NT-proBNP (median −535 pg/mL; IQR −1164.5, −218; *n* = 276; *P* < .0001) and hsTnI (median −3.4 ng/L; IQR −9.5, −0.9; *P* < .0001) were observed by week 12. These improvements were sustained with extended treatment at both weeks 48 and 96, respectively, for both NT-proBNP (−592 pg/mL; IQR −1352, −254; *P* < .0001; and −404.5 pg/mL; IQR −1371.5, −176.5; *P* < .01) and hsTnI (−5.0 ng/L; IQR −12.1, −1.0; *P* < .0001; and −5.2 ng/L; IQR −10.3, 0; *P* < .01) (*Figure 3*). Normal NT-proBNP (<150 pg/mL) was present in 10.6% of patients at baseline, increasing to 46.4%, 55.6%, and 65.9% at weeks 12, 48, and 96, respectively. Median percent decrease in NTproBNP was 79% at 96 weeks.

### Safety

Extended aficamten treatment was well tolerated in almost all patients with 98% of patients continuing therapy at the time of data cutoff. Six (2%) patients terminated treatment early: one due to ischaemic colitis, one who opted to proceed to surgical myectomy, one physician decision due to non-adherence, one after closure of their study site, one physician decision due to persistently elevated LVOT gradients despite 20 mg aficamten, and one physician decision due to baseline systolic dysfunction (this patient had multiple LVEF <50% events while on placebo in the parent study SEQUOIA-HCM). As of the data cutoff, four (1.4%) of these patients who terminated treatment had also discontinued from the study, and the other two completed an end-of-study visit shortly after data cutoff (both in September 2024). There were no deaths.

TEAEs were reported for 221 (74.7%) patients, of which the majority of TEAEs were mild or moderate in severity (*Table 2*). The most common adverse events were COVID-19, nasopharyngitis, and dizziness (*Table 3*). Eight (2.7%) patients experienced a TEAE leading to temporary interruption of aficamten (listed in *Table 2*). Atrial fibrillation occurred in 17 (5.7%) patients, seven (2.4%) of whom experienced new-onset atrial fibrillation (exposure-adjusted incidence rate [EAIR] of 2.0 per 100 patient-years). Amongst the 46 patients with a history of paroxysmal or persistent atrial fibrillation at the time of enrolment in FOREST-HCM, 10 experienced a TEAE of recurrent atrial fibrillation. Most atrial fibrillation events were mild or moderate in severity. None of the patients with new-onset atrial fibrillation developed systolic dysfunction (LVEF <50%) or heart failure, whereas one patient with a prior history of atrial fibrillation had an LVEF <50% event associated with alcohol-induced atrial fibrillation (see Supplementary data online, *Table S3*).^12^

**Table 2:** TEAEs and events of special interest

Event	Patients, *n* (%)	Patients with event per 100 patient-years
Patients with ≥1 TEAE	221 (74.7)	–
Patients with ≥1 mild TEAE	107 (36.1)	–
Patients with ≥1 moderate TEAE	87 (29.4)	–
Patients with ≥1 severe TEAE	27 (9.1)	–
Patients with TESAEs	36 (12.2)	–
Patients with fatal TEAEs	0 (0)	–
Patients with TEAEs leading to drug interruption^a^	8 (2.7)	–
Patients with TEAEs leading to dose reduction^b^	8 (2.7)	–
Patients with AEs related to study drug (per investigator)	40 (13.5)	–
LVEF <50%^c^	10 (3.4)	2.9
LVEF <50% associated with TEAE^d^	3 (1.0)	0.9
LVEF <50% associated with TESAE of signs or symptoms of heart failure	0	0
New-onset atrial fibrillation	7 (2.4)	2.0
Recurrent atrial fibrillation	10 (3.4)	2.9
Ventricular arrhythmias requiring treatment	1 (0.3)	0.3
Appropriate ICD discharge	1 (0.3)	0.3
Heart failure leading to hospitalization ^e^	1 (0.3)	0.3

^a^TEAEs leading to temporary interruption included atrial fibrillation (3 patients), COVID-19 infection, diarrhoea, hypertensive urgency, chest pain and dyspnoea, and mechanical fall resulting in a subdural hematoma and craniofacial fracture.

^b^Additional TEAEs leading to dose reduction included an erroneously calculated prolonged QT interval as reported previously,^12^ abdominal discomfort (ischaemic colitis), migraine, headache, dyspnoea, and gastrointestinal dysfunction.

^c^Events of LVEF <50% were associated with dose reduction.

^d^Associated TEAEs were dyspnoea (2 patients) and decreased ejection fraction (1 patient) and classified as mild or moderate.

^e^Acute on chronic diastolic heart failure exacerbation.

AE, adverse event; LVEF, left ventricular ejection fraction; TEAE, treatment-emergent adverse event; TESAE, treatment-emergent serious adverse event.

**Table 3:** TEAEs reported in ≥5% of patients

TEAE	Patients, *n* (%)	Patients with event per 100 patient-years
COVID-19	39 (13.2)	12.5
Nasopharyngitis	23 (7.8)	6.8
Dizziness	22 (7.4)	6.7
Dyspnoea	20 (6.8)	5.9
Palpitations	20 (6.8)	5.9
Headache	19 (6.4)	5.8
Upper respiratory tract infection	19 (6.4)	8.1
Hypertension	17 (5.7)	3.6
Atrial fibrillation	17 (5.7)	5.1
Recurrent	10 (3.4)	2.9
New onset	7 (2.4)	2.0
Fall	16 (5.4)	4.8
Arthralgias	15 (5.1)	4.5

TEAE, treatment-emergent adverse event.

Down-titration of aficamten was uncommon. Eight patients underwent dose reduction due to TEAEs (reported adverse events), all of which were mild or moderate in severity. Two down-titrations for TEAEs were due to an LVEF <50% (47%–49%), of which one event was in the same patient with alcohol-induced atrial fibrillation described previously.^12^ The other causes of dose reduction can be found in *Table 2*. Per protocol, down-titration due to LVEF <50% occurred in 10 (3.4%) patients (EAIR of 2.9 per 100 patient-years), of which two were reported as a TEAE (noted above); none had LVEF <40%. Amongst the 10 patients down-titrated due to LVEF <50%, eight were asymptomatic and the remaining two had mild symptoms (non-serious adverse events): one patient with dyspnoea shortly after receiving both flu and COVID-19 vaccines and the other patient with oedema (the same patient described above who was early terminated by physician decision due to underlying systolic dysfunction with multiple LVEF <50% readings on placebo while participating in SEQUOIA-HCM) (see Supplementary data online, *Table S3*).

TESAEs occurred in 36 (12.2%) patients (11.2 persons with event per 100 person-years), 10 (3.4%) of whom (2.9 persons with event per 100 patient-years) had a cardiac-related TESAE, none of which were deemed related to aficamten by the investigator. Cardiac TESAEs included atrial fibrillation in eight (2.7%) patients, acute myocardial infarction in two (0.7%) patients, embolic stroke (not related to atrial fibrillation) in two (0.7%) patients, and hospitalization for acute on chronic diastolic heart failure in one (0.3%) patient. Other TESAEs occurring in more than one patient are presented in *Table 4*. No patients had TESAEs of systolic dysfunction or severely reduced ejection fraction (LVEF <40%).

**Table 4:** Other TESAEs occurring in >1 participant

TESAE	Patients, *n* (%)	Patients with event per 100 patient-years
Fall	4 (1.4)	1.1
Embolic stroke	2 (0.7)	0.6
Road traffic accident	2 (0.7)	0.6
Syncope	2 (0.7)	0.6

TESAE, treatment-emergent serious adverse event.

## Discussion

Here we present comprehensive safety and efficacy data from FOREST-HCM in participants with symptomatic oHCM treated with aficamten comprising over 350 patient-years of exposure. Clinically significant and sustained improvements in symptoms, health status, haemodynamics, and biomarkers were observed with aficamten treatment. Consistent with prior reports,^6,7,12^ these improvements were achieved with modest overall LVEF reductions and with a low incidence of reversible and mostly asymptomatic LVEF <50% events with no associated heart failure hospitalizations, and without the need for temporary interruption in order to safely down-titrate the dose. Importantly, the incidence of new-onset atrial fibrillation remained low and was clinically mild when it occurred (*Structured Graphical Abstract*).

The population studied here is comprehensive, representing the majority of patients with oHCM who had completed a prior aficamten clinical trial (REDWOOD-HCM, SEQUOIA-HCM, or MAPLE-HCM), 98% of whom remained on treatment through the last follow-up. This may, in part, result from the magnitude of clinical response. Early and sustained mean reductions in resting and Valsalva LVOT gradients (∼50% with resting and Valsalva LVOT gradients <30 mmHg at week 12) were coupled with marked symptom improvement as measured by NYHA functional class (∼70% experienced improvement of at least one NYHA class by week 12) and KCCQ-CSS (>30% experiencing a large improvement). With continued aficamten treatment beyond week 12, a greater proportion of patients had resolution of LVOT obstruction and symptoms (NYHA class I), suggesting patients may accrue additional benefit with longer treatment. Mirroring these clinical improvements, reductions in cardiac markers of wall stress (NT-proBNP) and myocyte injury (hsTnI) were significant, sustained, and also continued to improve throughout extended treatment.

The combined impact of haemodynamic and symptom improvements is notable. By week 12 nearly a quarter of patients had a complete response with Valsalva LVOT gradient < 30 mmHg and NYHA class I symptoms, with the proportion of patients experiencing a complete response increasing subsequently at weeks 48 and 96 (40.5% and 62%). For comparison, and acknowledging lack of head-to-head comparison for mavacamten and aficamten, 46.8% of patients were complete responders to mavacamten within MAVA-LTE (A Long-Term Safety Extension Study of Mavacamten in Adults Who Have Completed MAVERICK-HCM or EXPLORER-HCM, NCT03723655).^13^ At baseline, 101 (34.1%) patients were guideline-eligible for SRT (NYHA class III and LVOT gradient ≥50 mmHg). Of note, one patient terminated therapy and underwent septal myectomy and another terminated therapy due to persistently elevated LVOT gradients despite 20 mg aficamten at weeks 52 and 36, respectively. There were three patients who did not have improvement in LVOT gradients or symptoms at the time of last follow-up, all of whom had transient improvement in Valsalva LVOT gradients and NT-proBNP, are on 20 mg aficamten, and remain in the study. This suggests that assessment of response should potentially include other metrics such as NT-proBNP and that a subset of patients might benefit from a higher dose. Of those who continued treatment with aficamten, only three (1%) patients remained eligible for SRT at week 12 and no one at week 72. When performed in a high-volume centre, SRT is associated with relief of LVOT obstruction, low procedural mortality, and substantial improvement in symptoms for 95% of patients^14,15^; however, patient access to these centres remains variable. Additionally, although SRT may be viewed as a ‘one-and-done’ treatment for patients with oHCM, patients who underwent SRT still require ongoing care and may develop symptoms, heart failure or arrhythmias, including atrial fibrillation, during follow-up. For instance, a recent report from the SHaRe (Sarcomeric Human Cardiomyopathy) Registry found that in patients who underwent SRT, new-onset atrial fibrillation occurred in 21% of patients, and 12% developed LVEF <50% after a mean of 6.8 years of follow-up.^16^ Acknowledging differences in patient population and length of follow-up, this provides context for the safety and efficacy of aficamten.

Owing to the mechanism of action of cardiac myosin inhibitors, assessment and monitoring of LVEF have become an important focus of patient safety. Ten (3.4%) patients were observed to have LVEF <50%, and none had LVEF <40%. Only 2 of the 10 patients had signs or symptoms (non-serious adverse events) at the time of the observed reduced LVEF, and LVEF reductions were reversible with non-interrupted down-titration of aficamten dosing. These findings are consistent with those observed in the Phase 2 and 3 placebo-controlled aficamten clinical trials and reflect a stable profile with extended exposure.^6,7^ In comparison, acknowledging aficamten and mavacamten have not been directly compared in clinical trials, the incidence of LVEF <50% for patients treated with mavacamten was reported to be 8.7% (EAIR 2.77 per 100 patient-years) within MAVA-LTE.^13^ Notably, dose titration in FOREST-HCM is based on site-read assessments of LVEF and LVOT gradients and incorporates investigator clinical judgement, suggesting these findings may be generalizable to real-world settings.

Per current European Society of Cardiology^2^ and American Heart Association/American College of Cardiology guidelines,^1^ the addition of a cardiac myosin inhibitor or disopyramide (or consideration for SRT) to first-line therapy (beta-blocker or non-dihydropyridine calcium channel blocker) is currently recommended for patients with oHCM who remain symptomatic despite maximally tolerated therapy. These guidelines recommend a patient-centred approach to decisions regarding second-line therapy and a comprehensive discussion of risks and benefits of medical and invasive therapies. The availability of longer-term data on safety and efficacy is required for individual patient-centred decision-making. Thus, this report on the efficacy and safety of aficamten administration over an extended period in patients with oHCM adds to the body of knowledge that will ultimately help inform provider-patient discussions.

Topline results were recently released for MAPLE-HCM indicating a favourable outcome for aficamten vs metoprolol as monotherapy in symptomatic oHCM.^17^ Notably, patients from MAPLE-HCM were subsequently enrolled in FOREST-HCM and some were included in this report.

The observed incidence of new-onset atrial fibrillation carries an important morbid impact for patients with oHCM and is, therefore, of significant interest. Atrial fibrillation is common in HCM, with a previously reported incidence of 2% per year,^18^ and risk of atrial fibrillation has been associated with age, increased left atrial size, and presence of LVOT obstruction. For patients with oHCM, concomitant atrial fibrillation is associated with increased symptom burden and risk of thromboembolism, leading to the addition of anticoagulation therapy, potential interventions for rhythm control, and the associated risks of these interventions. In this context it is reassuring that we observed a similar EAIR of 2 per 100 patient-years for patients taking aficamten. For comparison, again acknowledging aficamten and mavacamten have not been directly compared in clinical trials, the EAIR for atrial fibrillation was reported as 4.5 per 100 patient-years reported from MAVA-LTE.^13^ In addition to reduction in LVOT gradients, patients treated with aficamten for 24 weeks in SEQUOIA-HCM^19,20^ were observed to have decreased left atrial size and improved diastolic function, leading to the hypothesis that, over extended follow-up, these patients may have a lower risk of new or recurrent atrial fibrillation.

This report builds upon previously reported data from FOREST-HCM^12^ and demonstrates sustained efficacy in reducing LVOT obstruction and improving symptoms for patients with symptomatic oHCM. This report also provides an evaluation of safety during extended treatment with aficamten, showing low incidences of LVEF <50% and new-onset atrial fibrillation, and no deaths.

There are a few important aspects of study design that should be noted. Dosing adjustments were not blinded, not mandated, and were made at the discretion of the physician on the basis of clinical context for patients eligible for dose increase. For instance, dose increases were not forced by a programmed drug dispense system but were subject to patient and physician judgements. Although this reflects real-world practice, it also may have resulted in some patients not reaching the highest effective dose. Patients eligible for this extension study previously completed a parent clinical trial which could have potentially led to selection bias. Patients completing one of three parent studies were eligible for enrolment in FOREST-HCM; however, FOREST-HCM was not initially available for patients completing REDWOOD-HCM leading to a longer period of time between completion of this parent study and enrolment in FOREST-HCM compared to patients completing SEQUOIA-HCM and MAPLE-HCM. This could have led to attrition for this symptomatic oHCM patient cohort due to choosing an alternative treatment option; however, the majority of patients chose to enrol in FOREST-HCM. Additionally, all patients were off aficamten or assigned investigational drug from the parent study for a minimum period of 4 weeks, which would have been sufficient to allow washout prior to enrolment in FOREST-HCM. Regardless, we observed significant and sustained improvements in LVOT gradients and symptom burden for patients treated with aficamten, as has been observed within placebo-controlled clinical trials. Though the number of patients who completed week 96 follow-up and beyond was relatively small, this study is ongoing with additional patients who will be eligible for enrolment upon completion of ongoing parent studies, which will add to the growing body of long-term safety and efficacy data.

In conclusion, patients with symptomatic oHCM with extended aficamten treatment (352 patient-years, median follow-up 51.6 weeks) were observed to have early, substantial, and sustained improvements in LVOT gradients and symptom burden, with low incidences of LVEF <50% or new-onset atrial fibrillation. There were no occurrences of LVEF <40%, no instances of LVEF <50% with associated heart failure, and no deaths. LVEF reductions were reversible with simple down-titration without the need for any dose interruption. FOREST-HCM mimics current clinical practice, including site-read echocardiograms and integration of clinical judgement in dose adjustment decisions. These findings support a favourable long-term safety and efficacy profile of aficamten.

## Supplementary Material

ehaf1085_Supplementary_Data

## Declarations

### Data Availability

Qualified researchers may submit a request containing the research objectives, endpoints/outcomes of interest, statistical analysis plan, data requirements, publication plan, and qualifications of the researcher(s). Requests are reviewed by a committee of internal and external advisers. If approved, data necessary to address the research question will be provided under the terms of the data sharing agreement. Requests will be considered after applications for marketing authorization in the USA and Europe have been reviewed and final decisions rendered. Requests may be submitted to medicalaffairs@cytokinetics.com.

### Pre-registered Clinical Trial Number

FOREST-HCM (NCT04848506).
