# Supplementary material for: Aficamten in symptomatic obstructive hypertrophic cardiomyopathy: the FOREST-HCM long-term study
Source: Eur Heart J. 2026 Mar 5;47(24):3120–31. doi: 10.1093/eurheartj/ehaf1085 (PMC13286636; doi:10.1093/eurheartj/ehaf1085)
Supplement: ehaf1085_Supplementary_Data [file ehaf1085_supplementary_data.pdf]

Supplementary Appendix
This appendix provides additional information for:

Efficacy and safety of extended treatment with aficamten in symptomatic obstructive hypertrophic cardiomyopathy in FOREST-HCM

Supplementary Figure 1. FOREST-HCM Study Design at time of this analysis

Supplementary Table 1. Sarcomeric Gene Mutations Observed

Supplementary Table 2. Clinical Profile of Subjects with Residual Valsalva LVOT Gradient and Symptoms on 20 mg Aficamten

Supplementary Table 3. Clinical Profile of Patients Who Experienced LVEF <50%

Supplementary Figure 1. FOREST-HCM Study Design at the Time of this Analysis

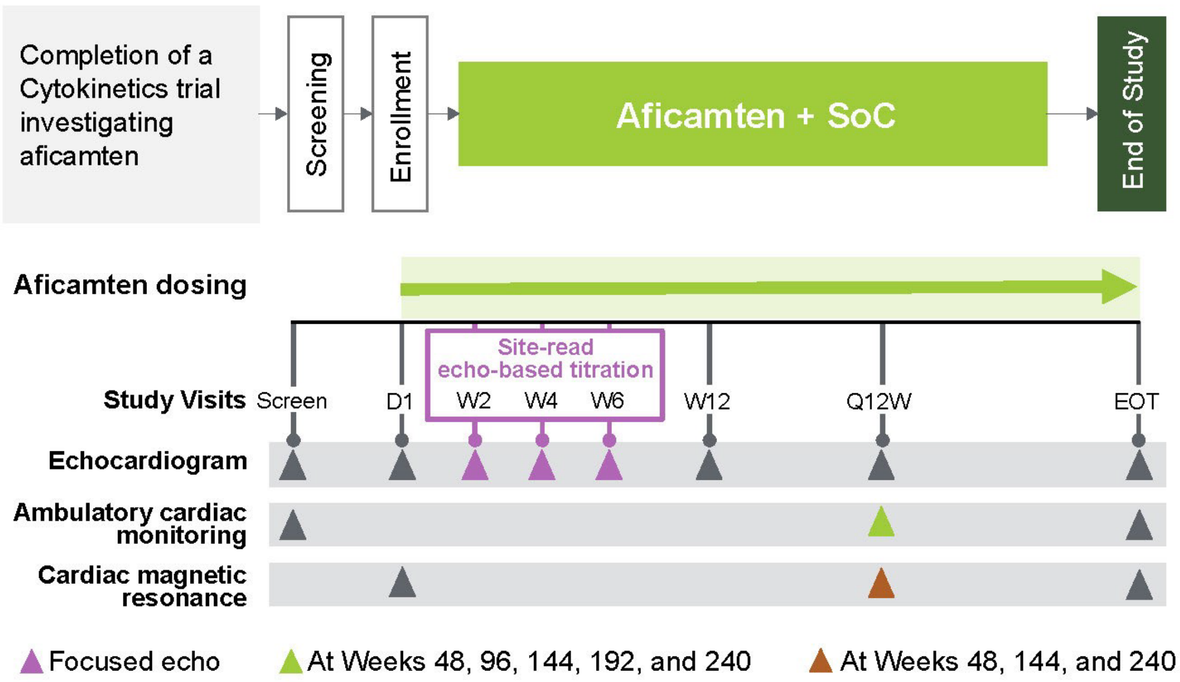

Note: Depicted is the study design at the time of this analysis. A subsequent protocol amendment has been approved allowing for greater flexibility in titration and less frequent monitoring.

Echo, echocardiography; EoT, end of treatment; D, day; Q12W, every 12 weeks; SoC, standard of care; W, week.

Reprinted from *JACC-HF*, Vol /edition number, Saberi et al., Aficamten Treatment for Symptomatic Obstructive Hypertrophic Cardiomyopathy: 48-weeks Results From FOREST-HCM, 2025 Aug, 13 (8) 102496, with permission from Elsevier

Supplementary Table 1. Sarcomeric Gene Mutations Observed

	**Patients, *n* (%)**
Family History of HCM	76 (25.7)
Sarcomeric Gene Mutation*	71 (93.4)
MYBPC3	42 (55.3)
MYH7	21 (27.6)
MYL2	2 (2.6)
MYL3	1 (1.3)
ACTC	0
TNNT2	5 (6.6)
TNNI3	2 (2.6)
TPM1	2 (2.6)

*: Percentages based on patients with available genetic data; patients with multiple sarcomeric mutations were counted in multiple categories.

Supplementary Table 2. Clinical Profile of Subjects with Residual Valsalva LVOT Gradient and Symptoms on 20 mg Aficamten

Subject	Background Therapy	History of Afib	Study Visit	Dose (mg)	NYHA	Resting LVOT Gradient (mmHg)	Valsalva LVOT Gradient (mmHg)	LVEF (%)	KCCQ-CSS	NTproBNP (pg/mL)	hsTroponin I (ng/L)
76M	none	no	Day 1	0	Class II	91	190	77	92	629	16
			Week 12	20	Class II	13	67	65	92	151	9
			Week 24	20	Class II	106	190	80	83	181	15
35M	Beta Blocker	no	Day 1	0	Class I	37	87	82	49	1606	9
			Week 12	20	Class I	32	68	85	78	401	9
			Week 24	20	Class I	57	73	69	74	238	11
			Week 36	20	Class I	65	100	81	75	922	11
52M	Non-DH CCB	yes	Day 1	0	Class II	66	86	70	70	1079	1000
			Week 12	15	Class II	12	42	57	65	488	499
			Week 24	20	Class II	36	90	65	65	486	591
			Week 36	20	Class II	75	100	65	47	574	766

PK data was not available to confirm compliance. Non-DH CCB: non-dihydropyridine calcium channel blocker

Supplementary Table 3. Clinical Profile of Patients Who Experienced LVEF <50%

							At Low EF							Latest Follow-up			
Pt ID	Age, y	Sex	AFI dose at low EF, mg/d	Bkgr tx	BL LVEF %	Study week, LVEF <50%[a]	LVEF %	VALS LVOT-G, mmHg	NYHA class	KCCQ-CSS [b]	Absolute NT-proBNP (Δfrom BL), ng/dL[b]	Assoc. AE, severity	Next visit LVEF, %	Study week	LVEF %	AFI dose, mg/d	Perm. Disc
1	45	M	15	BB	69	8	47	7	II	NA	NA	Alcoh-induc'd AFib	60.2	132	67	10	No
2	60	M	20	CCB+ DISO	67	48	49	16	I	98	80 (−729)	none	55	132	52	15	No
3	74	F	15	BB	65	72	45	4	I	95	119 (−614)	none	55	108	55	10	No
4	57	M	20	BB	61	60	47	31	I	NA	103 (−243)	none	54	132	63	15	No
5	77	F	15	BB	66	12	49	22	III	66.46	229 (91)	Dyspn[c]	42	48	60	5	No
6	75	F	10	FURO	55	6	45	112	III	NA	NA	Dyspn[d]	52	20	42	NA	Yes
7	41	M	15	DISO	56	38	41	12	II	NA	614 (−817)	None	57	36	60	10	No
8	65	M	15	BB	63	24	43	6	II	82.29	<4.9 (−216)	None	52	48	69	10	No
9	66	M	20	CCB	65	8	47	28	II	NA	NA	None	55	36	55	5	No
10	71	F	20	BB	66	36	49.9	8	I	96	139 (−1300)	None	57	38	57	15	No

a For patients with multiple LVEF <50% events (pt IDs 4, 6, and 9), only data from the first event is listed.

b KCCQ and NT-proBNP were collected at prespecified study visits per protocol and thus not available for every time point.

c Occurred following both COVID and flu shots.

d Multiple LVEF<50% events on PBO in SEQUOIA-HCM.

AE, adverse event;; AFI, aficamten; Alcoh-induc'd AFib, alcohol-induced atrial fibrillation; Assoc, associated; BB, beta-blocker; Bkgr tx, background treatment; BL, baseline; CCB, calcium channel blocker; Disc, discontinued; DISO, disopyramide Dyspn, dyspnoea; EF, ejection fraction; F, female; FURO, furosemide; KCCQ-CSS, Kansas City Cardiomyopathy Questionnaire Clinical Summary Score; LVEF, left ventricular ejection fraction; LVOT-G, left ventricular output tract gradient; M, male; NA, not available; NT-proBNP, N-terminal pro–brain natriuretic peptide; NYHA, New York Heart Association; PBO, placebo; Perm, permanent; pt ID, patient identifier (masked); VALS, Valsalva.
